# The correlation between anorexia nervosa and childhood traumatic experience: the mediating role of impulsivity

**DOI:** 10.1186/s40337-026-01557-2

**Published:** 2026-02-26

**Authors:** Jing Zhang, Yanran Hu, Qing Kang, Mengting Wu, Yunling Zou, Sufang Peng, Jue Chen

**Affiliations:** 1 Department of Psychological Medicine, Shanghai Xuhui District Dahua Hospital, Shanghai, China; 2 Department of Clinical Psychology, Shanghai Mental Health Center, Shanghai Jiao Tong University School of Medicine, Shanghai, China; 3 Shanghai Changning Mental Health Center, Shanghai, China

**Keywords:** Anorexia nervosa, Childhood traumatic experience, Emotional abuse, Impulsivity, Mediating role

## Abstract

**Background:**

The pathogenesis of anorexia nervosa (AN) involves multiple factors, among which childhood traumatic experience has attracted attention. Emotional abuse, as a form of trauma, may exert a predictive effect on AN. In addition, childhood traumatic experience is closely linked to impulsivity, yet the trauma-impulsivity-AN mediation pathway has not been directly validated. The purpose of this study is to compare differences in childhood traumatic experience of patients with different subtypes of AN, as well as exploring the mediating role of impulsivity between emotional abuse in childhood traumatic experience and symptom severity of AN.

**Methods:**

This study included 157 female patients with AN, including 76 with the restricting type (AN-R) and 81 with the binge-eating/purging type (AN-BP), as well as 124 matched healthy controls (HC). Childhood traumatic experience was evaluated using Early Trauma Inventory-short form (ETI-SF), impulsivity assessed by Barratt Impulsiveness Scale 11th Version (BIS-11), and clinical characteristics via Eating Disorder Examination-questionnaire (EDE-Q 6.0), Beck Depression Inventory (BDI) and Beck Anxiety Inventory (BAI). Furthermore, inter-group differences in childhood traumatic experience were determined by one-way analysis of variance and analysis of covariance, the correlation between childhood traumatic experience and AN by Pearson correlation analysis, and the mediating role of impulsivity between emotional abuse and symptom severity was clarified by the Bootstrap method.

**Results:**

Cases in the AN-BP group had significantly higher exposure to childhood traumatic experience compared with the AN-R and HC groups (both *p* < 0.05). Significant differences were likewise observed in emotional-abuse scores across the three groups (*F* = 10.574, *p*= 0.000, partial *η*² = 0.084). Emotional abuse was positively correlated with impulsivity and symptom severity of AN (both *p* < 0.05). In addition, the mediation effect of impulsivity between emotional abuse and symptom severity was 0.073 (95% CI 0.013 ~ 0.153), with an effect proportion of 19.363%.

**Conclusion:**

AN-BP patients have more significant childhood traumatic experience than AN-R patients, and difference was mainly reflected in the emotional abuse factor. Emotional abuse has established correlation with impulsivity and symptom severity in AN, with impulsivity playing a mediating role between emotional abuse and symptom severity.

**Supplementary Information:**

The online version contains supplementary material available at 10.1186/s40337-026-01557-2.

## Abstract (plain language summary)

In recent years, the incidence rate of AN in China has been rising, drawing significant attention. Among the various causes, childhood traumatic experiences are closely linked to AN. In this study, we explored the correlation between childhood traumatic experience and AN. We found that AN patients with symptoms of binge eating and purging often suffered more traumatic experience (especially emotional abuse) in their childhood. We also found that impulsivity may act as a “bridge” between childhood trauma and AN. Through this study, we aim to elucidate the pathogenic pathway of AN and generate novel insights for its clinical intervention. We also hope this story invites every reader to look closer at children’s inner worlds—shielding them from emotional harm and letting growth unfold without scars.

**Supplementary Information:**

The online version contains supplementary material available at 10.1186/s40337-026-01557-2.

## Background

Anorexia nervosa (AN) is a psychiatric disorder defined by markedly low body weight, intense weight-gain fear, and distorted body image [1]. Clinically, it is divided into two subtypes based on behavioral manifestations: the restricting type (AN-R) and the binge-eating/purging type (AN-BP). AN-R patients maintain abnormally low body weight through strict dietary restriction, whereas AN-BP patients exhibit recurrent binge-eating episodes accompanied by purging behaviors—such as self-induced vomiting, misuse of laxatives, or compulsive excessive exercise [2]. In terms of epidemiology, AN shows a marked gender disparity: the lifetime prevalence is as high as 4% in females, compared to only 0.3% in males [3]. The onset age is mostly concentrated in adolescence to young adulthood [4]. The clinical significance of AN lies in its high mortality rate and severe impact on patients’ quality of life. As one of the most life-threatening psychiatric disorders, AN not only causes severe psychological impairment but also leads to a wide range of somatic complications, including severe malnutrition, electrolyte imbalances, bradycardia, osteoporosis, endocrine dysfunction, and even multiple organ failure [5]. A study by Van et al. further confirmed that AN increases the risk of mortality by fivefold or more [3]. Among the two subtypes, AN-BP is associated with a higher likelihood of severe complications, comorbid mental health conditions, and suicidal behaviors—thus conferring a greater mortality risk than AN-R [6].

The development of AN is influenced not only by genetic and biological factors; numerous studies have also shown that traumatic experiences in childhood are significantly associated with greater illness severity, higher suicide risk, and more frequent binge–purge behaviors in AN [7, 8]. Trauma violates the physical and psychological boundaries of children, leading them to perceive their bodies as sites of pain controlled by external forces. In response, they attempt to regain a sense of control by refusing food or regulating intake. Throughout this process, the body becomes a battlefield where symbolism and physicality collide, and the relationship with food evolves into a core means of expressing and managing the pain caused by trauma [9]. A systematic analysis by Molendijk et al. found that AN is associated with all types of trauma including emotional, physical, and sexual abuse, and the severity of AN increases with cumulative trauma exposure [7]. However, a growing body of research suggests that emotional abuse exerts a more consistent and stronger predictive effect on AN compared with physical or sexual trauma [8–10]. Evidence suggests that seemingly inconspicuous factors, such as family environment disorganization and insufficient emotional support during development, may exert a potential influence on the risk of AN by inducing chronic psychological distress [11, 12]. Emotional abuse can disrupt an individual’s ability to accurately identify and adaptively regulate emotions, while also restricting the overt expression of affective states. These impairments in emotional processing are posited to be key mechanisms that predispose individuals to develop maladaptive eating patterns [13, 14]. Therefore, emphasizing emotional abuse as a central factor in the relationship between AN and childhood trauma is essential. Moreover, the majority of trauma-focused studies have examined eating disorders or AN as a single entity [8, 9], while comparisons of trauma profiles across AN subtypes remain scarce. Consequently, the present study places special emphasis on subtype differences in trauma exposure, aiming both to uncover distinct psychopathological mechanisms and to identify precise targets for tailored interventions.

A growing literature points to a link between childhood maltreatment and impulsive traits, although the underlying neurobiological and cognitive pathways remain elusive [15–17]. While AN patients have traditionally been characterized by “excessive control”, a growing body of evidence indicates that significant impulsive traits are also prevalent among this population—with such traits being particularly common in the AN-BP [2, 18, 19]. Although no existing studies have directly validated the trauma-impulsivity mediational model specifically in AN, relevant research provides indirect support for this framework. For instance, in a study of 102 patients with eating disorders, Corstorphine et al. observed that childhood trauma histories were linked to impulsive behaviours, which suggests that impulsivity may act as a “bridge” between childhood trauma and AN [20].

In view of this, the present study was designed to compare discrepancies in childhood traumatic experiences across patients with distinct subtypes of AN and to investigate the associations among childhood trauma, impulsivity and AN. We proposed two key hypotheses: first, patients with the AN-BP would report a higher prevalence of childhood traumatic experiences than those with the AN-R, and such differences might vary across different categories of trauma; second, impulsivity was hypothesized to exert a significant mediating effect in the relationship between childhood traumatic experiences and the symptom severity of AN.

## Methods

### Participants

**Fig. 1:**
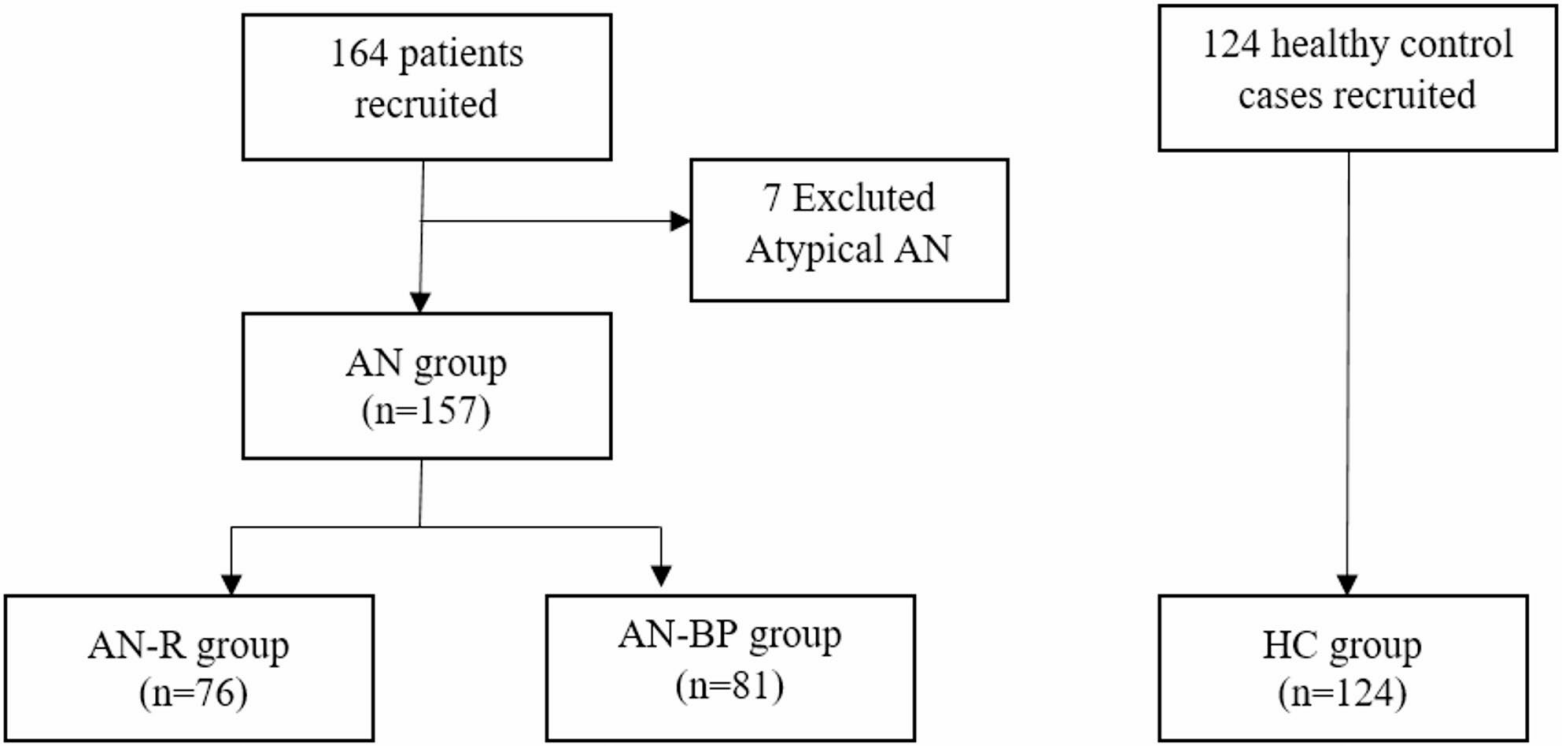
The flowchart of participants eligible for current study

From September 2019 to August 2024, 164 female patients were recruited from the Psychosomatic Medicine Ward and the Psychological Counseling Clinic of the Shanghai Mental Health Center. Following independent diagnostic confirmation by two senior psychiatrists, seven patients were excluded because they met criteria for atypical anorexia nervosa. As shown in Fig. 1, the final sample comprised 157 patients with AN, including 76 with AN-R and 81 with AN-BP (see Fig. 1).

Given the marked female predominance in the epidemiology of anorexia nervosa, with lifetime prevalence rates reported to be up to 4% in females compared with approximately 0.3% in males, only female participants were recruited in this study [3]. All participants were treatment-naive individuals aged 13–30 years who had never used psychotropic medication. This age range captures the peak incidence period of AN in females: 13 years represents the earliest common onset age while ensuring adequate comprehension of questionnaires, whereas 30 years retains high prevalence in early adulthood yet minimizes confounding comorbidities.

The healthy control (HC) group included 124 cases from the recruited population and students of Shanghai Jiao Tong University.

### Inclusion and exclusion criteria

The inclusion criteria for AN group were as follows: female; Han Chinese; aged 13–30 years; confirmed diagnosis of AN by two senior psychiatrists according to DSM-5 criteria; and body mass index (BMI) ranging from 13 to 18.5 kg/m2. Patients were further classified into AN-R or AN-BP based on the presence or absence of recurrent binge-eating or purging behaviors, including self-induced vomiting or misuse of laxatives, diuretics, or enemas. AN-R was defined by the absence of such behaviors, with low body weight maintained through dieting, fasting, or excessive exercise, whereas AN-BP was defined by the presence of these behaviors. The exclusion criteria for AN group were as follows: met the diagnostic criteria of other mental disorders except AN; serious passive suicidal ideation or behaviors; serious somatic diseases or physical complications; pregnant or breastfeeding.

The inclusion criteria for HC group were as follows: females; Han Chinese; aged 13–30 years; and BMI ranging from 18.5 to 23.9 kg/m2. The exclusion criteria for HC group were as follows: met any of the diagnostic criteria, as assessed using the Mini-International Neuropsychiatric Interview (M.I.N.I.) administered by trained psychiatrists; serious somatic diseases or physical complications; pregnant or breastfeeding.

### Demographic and clinical data

General information of all the enrolled participants was acquired by using a self-designed general information questionnaire, including study number, name, gender, age, occupation, marriage, education level, height, weight, BMI, contact information, history of major illnesses, history of suicide attempts and history of pregnancy. For AN patients, additional data were also included, such as the age at onset, total course of disease, precipitating factors, current diagnosis (including subtype), outpatient number, or hospitalization number.

### Assessment scales

#### Eating disorder examination-questionnaire (EDE-Q 6.0)

THE EDE-Q was adapted from the EDE by Fairburn and Beglin in 1994 [21]. EDE-Q 6.0 is a self-rating scale with 28 entries, which is divided into four subscales, including dietary restriction, eating concern, shape concern, and weight concern. Each item is rated on a 7-point scale, ranging from 0 (none at all or not at all) to 6 (every day or marked). The score for each subscale is calculated as the average of its respective items, reflecting the severity of symptoms in that particular domain. The total scale score, which indicates the overall severity of eating disorder symptoms, is derived by averaging the sum of the four subscale scores. Higher scores on both the subscales and the total scale denote greater symptom severity. The Chinese version of EDE-Q 6.0 has good psychometric properties and diagnosis accuracy in mainland Chinese female patients with eating disorders (Cronbach’s *α* = 0.91) [22]. In this study, EDE-Q 6.0 was employed to assess the clinical behavioral and psychological characteristics of AN, and to assess the severity of AN-specific clinical symptoms.

#### Barratt impulsiveness scale 11th Version (BIS-11)

BIS was originally developed by American scholar Dr. Barratt in 1959. Since then, it has undergone 11 revisions, with the current version being the BIS-11 [23]. BIS-11 is a self-rating scale with 30 items and includes three dimensions: attentional impulsiveness, motor impulsiveness, and non-planning impulsiveness. Each item is scored on a 4-point scale (rarely/never = 1, occasionally = 2, often = 3, almost always/always = 4) with some items being reverse-scored. The total score ranges from 30 to 120, with higher scores indicating higher levels of impulsivity. The Chinese version of BIS-11 has good reliability and validity, and it is the most commonly used self-report scale for measuring impulsivity (Cronbach’s *α* = 0.75) [24]. In this study, it was used to assess participants’ impulsive personality traits.

#### Early trauma inventory-short form (ETI-SF)

ETI-SF was originally developed by J. Douglas Bremner and colleagues in 2007 [25]. This self-rating scale has 27 items covering four dimensions of general trauma, physical trauma, emotional abuse, and sexual trauma. Each item has two options of “yes” and “no”, with “yes” scoring 1 point and “no” scoring 0 point. The ETI-SF is widely used to assess an individual’s traumatic experiences during childhood due to its brevity and effectiveness. In this study, the ETI-SF was chosen as the assessment tool with the aim of quickly and accurately identifying the participants’ childhood traumatic experiences. The Chinese version of this questionnaire has been validated to possess good reliability and validity (Cronbach’s *α* = 0.83) [26].

#### Beck depression inventory-II (BDI-II)

BDI-II was developed by Beck in 1996 [27]. It is a self-rating scale including 21 items. Each item is rated on a 4-point scale, ranging from 0 to 3 (0 = “not at all” and 3 = “severe”). The higher the total score of the scale, the more severe depression symptoms. BDI-II was employed to assess participants’ depressive symptoms in the present study. The Chinese version of BDI-II has good reliability and validity (Cronbach’s *α* = 0.94) [28].

#### Beck anxiety inventory (BAI)

BAI was originally developed by Beck and his collaborators in 1985 [29]. It is a self-rating scale including 21 items. Respondents are asked to rate each item on a 4-point scale of 0 to 3 (0 = “not at all” and 3 = “severe”). Item scores are summed to produce a total ranging from 0 to 63, with higher scores reflecting greater anxiety severity. The Chinese version of BAI has demonstrated excellent internal consistency among mainland Chinese patients (Cronbach’s *α* = 0.95) [30].

### Statistical analyses

Database establishment and data analysis were completed in SPSS 26.0. Measurement data were evaluated for normality by Kolmogorov-Smirnov normality test and stem-and-leaf plot, with normally distributed data expressed as x ± s. Inter-group and multi-group comparisons employed independent samples t-test, and one-way analysis of variance, respectively, and pairwise comparison adopted the LSD method. Covariates were then controlled for using analysis of covariance (ANCOVA). Pearson correlation analysis was utilized to profile the correlations of continuous variables. Finally, the percentile Bootstrap method (PROCESS plug-in) was used to perform mediation effect test [31], with the random sampling set as 5,000 times and reported 95% confidence interval (CI). The presence of statistically significant difference was determined when *p* < 0.05.

## Results

### Comparison of childhood traumatic experience

Inter-group comparison revealed no statistically significant differences in age, height and years of education between AN and HC groups (all *p* > 0.05), with BMI of the AN group significantly lower than the HC group (15.27 ± 2.22 kg/m2 vs. 19.76 ± 2.41 kg/m2, *p* < 0.05). Meanwhile, the AN-R group had lower BMI than the AN-BP group (14.40 ± 2.26 kg/m2 vs. 16.22 ± 1.78 kg/m2, *p* < 0.05), and the AN-R group was significantly younger than the AN-BP group (16.40 ± 3.22 vs. 19.94 ± 4.89, *p* < 0.05).

According to the comparison of childhood traumatic experience among three groups, a one-way ANOVA revealed a significant main effect of groups on the ETI-SF total score (*F* = 6.203, *p* = 0.002, partial *η*² = 0.053). Pairwise comparisons indicated that the AN-BP group had significantly higher ETI-SF total score than those of the AN-R and HC groups (*p* < 0.05). Significant differences were likewise observed in emotional-abuse scores across the three groups (*F* = 10.574, *p* = 0.000, partial *η*² = 0.084). The AN-BP group had significantly higher emotional abuse scores than those of the AN-R and HC groups (both *p* < 0.05). In addition, the general trauma score in the AN-R group was significantly lower than HC groups (*p* < 0.05), but no significant difference was observed between the AN-R and AN-BP groups (*p* = 0.071). Furthermore, there was no significant statistical difference in the scores of physical trauma and sexual trauma among the three groups (all *p* > 0.05). However, the average score of sexual trauma in the AN-BP group was higher than that in the other two groups (see Table 1).

After controlling for BMI and age with analysis of covariance (ANCOVA), the statistically significant differences among the three groups in ETI total scores and in physical, emotional and sexual trauma remain consistent with the previous results; only the significant difference in general trauma between the HC and AN-R groups disappeared. This suggests that age and BMI partly accounted for the initial group difference. Considering that older age is associated with greater cumulative exposure to adverse experiences and that lower BMI potentially affects memory or emotion states related to trauma recall, the original difference in general trauma may be attributable to these confounding factors rather than to group status alone. Details are provided in Supplementary Table 1.

**Table 1:** Comparison of childhood traumatic experience between AN-R group, AN-BP group, and HC groups

	AN-*R* (*n* = 76)	AN-BP (*n* = 81)	HC (*n* = 124)	F	*p*	partial η²	Pairwise comparison *p*-values		
							AN-*R* Vs AN-BP	AN-*R* Vs HC	AN-BP Vs HC
ETI-SF Total score	3.27± 3.01	5.64± 4.29	4.20± 3.52	6.203	0.002	0.053	0.000	0.082	0.017
General trauma	0.59± 1.16	1.09± 1.67	1.21± 1.56	3.643	0.028	0.032	0.071	0.008	0.613
Physical trauma	1.37± 1.52	1.89± 1.79	1.56± 1.42	1.780	0.171	0.016	0.063	0.424	0.182
Emotional abuse	1.11± 1.47	2.29± 1.89	1.24± 1.45	10.574	0.000	0.084	0.000	0.593	0.000
Sexual trauma	0.15± 0.44	0.38± 0.98	0.19± 0.57	1.922	0.149	0.017	0.069	0.689	0.096

### Correlation analysis between AN and childhood traumatic experience

According to the correlation analysis, the ETI-SF total score revealed significantly positive correlations with EDE-Q 6.0 total score, BDI score and BAI score (*r* = 0.249–0.350, *p* = 0.000-0.006). Furthermore, emotional abuse in the ETI-SF scale also yielded significantly positive correlations with EDE-Q 6.0 total score, BDI score and BAI score (*r* = 0.401–0.471, *p* = 0.000). Physical trauma was significantly positively correlated with BDI score and BAI score (*r* = 0.282, *p* = 0.002; *r* = 0.257, *p* = 0.005). However, general trauma and sexual trauma in the ETI-SF scale had no obvious correlations with clinical characteristics of AN (all *p* > 0.01) (see Table 2).

The correlation analysis in Table 2 also showed that the ETI-SF total score exhibited significantly positive correlations with the BIS-11 score (*r* = 0.240, *p* = 0.014). Meanwhile, emotional abuse in the ETI-SF scale was significantly and positively correlated with the BIS-11 score (*r* = 0.285, *p* = 0.003). However, no significant correlation was observed in general trauma, physical trauma, and sexual trauma with BIS-11 score (*p* > 0.05).

**Table 2:** Correlation between AN and childhood traumatic experience

		ETI-SF total score	General trauma	Physical trauma	Emotional abuse	Sexual trauma
EDE-Q 6.0 total score	Pearson correlation	0.249**	0.058	0.140	0.409^**^	-0.002
	Significance (bilateral)	0.006	0.530	0.127	0.000	0.987
BDI score	Pearson correlation	0.290**	-0.012	0.282**	0.401**	0.053
	Significance (bilateral)	0.002	0.898	0.002	0.000	0.569
BAI score	Pearson correlation	0.350**	0.018	0.257**	0.471**	0.194
	Significance (bilateral)	0.000	0.845	0.005	0.000	0.034
BIS-11 score	Pearson correlation	0.240^*^	0.103	0.149	0.285^**^	0.091
	Significance (bilateral)	0.014	0.295	0.127	0.003	0.351

### Mediation effect test of impulsivity

Given the strong correlations of emotional abuse in childhood traumatic experience with impulsivity and symptom severity, Model 4 of the PROCESS plug-in was used for modeling, with emotional abuse as the independent variable, symptom severity as the dependent variable, and impulsivity as the mediator variable. Based on the results of regression analysis, emotional abuse indicated a significant positive predictive effect on impulsivity (*β* = 1.551, *t* = 2.995, *p* = 0.003) and symptom severity (*β* = 0.304, *t* = 4.123, *p* = 0.000), suggesting 0.304-unit increase in symptom severity per one-unit increase in emotional abuse. Moreover, after controlling for emotional abuse, there was still a significant direct effect of impulsivity on symptom severity (*β* = 0.047, *t* = 3.492, *p* = 0.001) (see Table 3).

Our study continued to investigate the mediation effect of impulsivity between emotional abuse and symptom severity in AN patients by employing a Bootstrap method. The results showed that the total effect of emotional abuse on symptom severity was 0.377, with a CI not containing 0 (95% CI 0.229 ~ 0.524). The direct effect of emotional abuse on symptom severity was 0.304, with the CI not containing 0 (95% CI 0.158 ~ 0.450). The mediation effect value of impulsivity between emotional abuse and symptom severity was 0.073, with the CI of Bootstrap not containing 0 (95% CI 0.013 ~ 0.153) and an effect proportion of 19.363%. As a result, impulsivity might exert a partial mediation effect between emotional abuse and symptom severity (see Table 4).

**Table 3:** Regression analysis of the mediation model

Variable	Dependent variable					
	Symptom severity		Symptom severity		Impulsivity	
	t value	*p*	t value	*p*	t value	*p*
Emotional abuse	4.123	0.000	5.066	0.000	2.995	0.003
Impulsivity	3.492	0.001				
*R*² value	0.287		0.201		0.081	
*F* value	20.336		25.660		8.971	

**Table 4:** Mediation effect analysis of impulsivity

Effect type	Effect value	SE	95%CI	Relative effect ratio/%
Total effect	0.377	0.074	0.229 ~ 0.524	100.000
Direct effect	0.304	0.073	0.158 ~ 0.450	80.637
Indirect effect	0.073	0.036	0.013 ~ 0.153	19.363

**Fig. 2:**
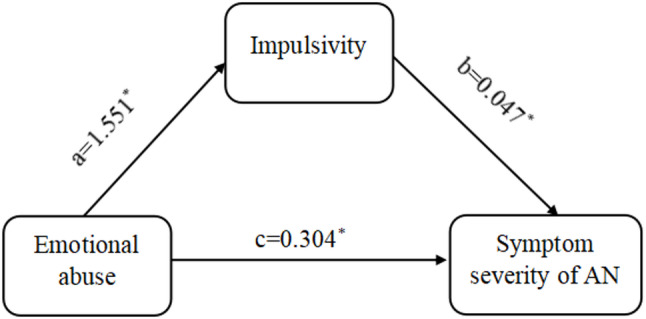
Mediation effect model of impulsivity. ^*^P < 0.05

## Discussion

The objective of this study is to compare the differences in childhood traumatic experiences across patients between distinct subtypes of AN. Our findings indicate that AN-BP patients reported more childhood traumatic experience than both AN-R and HC participants, which is consistent with our hypothesis. This argument also supports the study by Molendijk et al., who identified a significant association between childhood maltreatment and binge-eating/purging behaviors [7]. After comparing specific trauma forms, we found that the differences across subtypes were primarily driven by the emotional abuse factor. As the meta-analysis by Guillaume et al. shows, emotional abuse has a dose-response relationship with emotional dysregulation, symptom severity, and comorbid depression in patients with AN, and its effect size often exceeds that of physical or sexual abuse [8]. Our findings are also consistent with the cross-sectional study conducted by Jaite et al. They linked emotional abuse to impulsive/emotionally unstable traits in AN-BP and proposed that purging serves to down-regulate trauma-related affect [32]. However, after controlling for the potentially confounding effects of BMI and age, we found no statistically significant differences among the two AN subtypes and HC in terms of sexual or physical trauma—an outcome that diverges from previous reports indicating that individuals with AN have experienced higher rates of such trauma than the general population [33, 34]. Possible explanations include a genuine cohort reduction following societal and legal improvements, as well as under-reporting due to privacy concerns. It is worth noting that although the between-group difference in sexual trauma scores was not statistically significant, the mean score of the AN-BP group was nearly twice that of the AN-R and HC groups, implying a potentially higher experience of sexual trauma among AN-BP patients. These preliminary observations should be corroborated in adequately powered, large-scale studies. Finally, no significant differences in general trauma were reported across groups. However, relevant literature also notes that trauma tends to exhibit multiple cumulative effects, and major life stressors (such as car accidents, severe natural disasters, parental divorce, and serious illnesses, among others) may lead to other types of trauma [9]. To summarize, emotional abuse may play an important role in the pathogenesis of AN and its subtype differentiation, yet other types of trauma should not be overlooked.

On the basis of previous studies, it can be understood that AN-BP patients exhibited more significant impulsivity than AN-R patients, which was in line with the recurrent binge eating and purging behaviors [2]. In this study, childhood traumatic experience was positively linked to AN symptom severity, impulsivity, and anxiety-depression levels. Emotional abuse showed the strongest and most consistent associations, highlighting the unique salience of emotional abuse in the trauma–AN pathway. Consistent with our findings, Racine & Wildes likewise contend that childhood emotional abuse is the form of maltreatment most strongly tied to AN psychopathology, and it is related to impulsive behaviors when experiencing negative emotions [10].

As far as we know, this study is the first to explore the mediating role of impulsivity in the relationship between emotional abuse and the severity of AN. Consistent with our hypotheses, emotional abuse exerted both a direct effect on symptom severity and an indirect effect through heightened impulsivity traits. This mediated pathway identifies impulsivity as a critical psychological mechanism that may transmit the impact of early emotional maltreatment onto subsequent AN psychopathology (see Fig. 2). Evidence from non-clinical samples provides converging support: among young adult women, binge-eating behaviors were shown to be jointly influenced by childhood trauma and impulsivity [35], mirroring the mediated pathway identified in our clinical AN sample. Theoretically, these findings align with the emotion-dysregulation–impulsivity model articulated within dialectical behavior therapy. Emotional abuse is posited to erode the capacity for effective regulation of negative affect, thereby heightening susceptibility to dysregulated emotional states. When confronted with acute distress, individuals may resort to impulsive behaviors as expedient emotion-regulation strategies [36–38], ultimately contributing to the exacerbation of AN symptoms. By differentiating trauma subtypes and focusing on clinical AN populations, the present study extends prior research and clarifies the specific pathway linking emotional abuse, impulsivity, and AN severity.

Accordingly, it may provide us with some insights into clinical psychotherapy, where different psychological treatment methods can be used for targeted interventions on childhood traumatic experience of patients [39]. For example, family-based treatment can be employed to adjust family interaction mode, repair family relationships, and reduce family environment-induced emotional neglect or emotional abuse [40]. Dialectical behavior therapy targeting impulsivity may facilitate patients’ understanding and grasp of emotional regulation skills and mindfulness skills, thus dealing with emotional dysregulation and impulsive behaviors caused by childhood traumatic experience [36]. Additionally, cognitive behavioral therapy can be used to deal with traumatic memory; while gradual exposure and cognitive reconstruction can be utilized to reduce negative emotions and impulsive behaviors caused by traumatic experience [41]. We hope this study offers clinicians a fresh lens on AN, and more importantly, inspires society to safeguard children’s inner worlds with compassionate vigilance against emotional harm.

### Strengths and limitations

The present study has several strengths. Firstly, to our knowledge, it is the largest Chinese sample to date comparing childhood trauma profiles between AN-R and AN-BP subtypes with a standardized, validated questionnaire (ETI-SF). Secondly, we present the first study to construct and test a “childhood emotional abuse – impulsivity – symptom severity of AN” mediation model, thereby filling a critical research gap in the field. Finally, our findings offer a fresh mechanistic perspective and point to impulsivity and trauma as promising, modifiable targets for future clinical intervention in AN.

Nevertheless, several limitations should also be acknowledged. First, the sample size was determined by references from previous studies and clinical availability rather than an a-priori power analysis; consequently, we cannot rule out the possibility that the study was under-powered to detect smaller effects (e.g., sexual trauma differences). Second, the cross-sectional design precludes causal inferences; longitudinal or experimental work is needed to verify whether emotional abuse precedes impulsivity and, in turn, AN severity. Third, all measures were self-reported, potentially subject to recall and social-desirability biases; multi-informant or objective indicators would strengthen future studies. Fourth, we examined only one mediating pathway—impulsivity; other candidate mechanisms (e.g., emotion-regulation deficits, genetic polymorphisms, inflammatory markers) were not modelled. Last but not least, the sample was restricted to Han Chinese females aged 13–30 years; findings may not generalize to males, older adults, or other ethnic groups.

## Conclusion

In conclusion, AN-BP patients reported more prominent childhood traumatic experience in comparison to AN-R patients, which is particularly prominent in emotional abuse. Moreover, the study identified a significant association between emotional abuse and both impulsivity traits and the severity of clinical symptoms among AN patients. Importantly, impulsivity was found to serve as a mediating factor in the relationship between emotional abuse and the severity of AN symptoms—indicating that the link between emotional abuse and AN symptom severity involves an indirect pathway through impulsivity, in addition to any direct association.

## Supplementary Information

Below is the link to the electronic supplementary material.

Supplementary Material 1.

## Data Availability

The datasets used and analyzed during the current study are available from the corresponding author on reasonable request.

## Abbreviations

AN: Anorexia nervosa
AN-R: Restricting-type of anorexia nervosa
AN-BP: Binge-eating/purging type of Anorexia nervosa
HC: Healthy controls
ETI-SF: Early trauma inventory-short form
BIS-11: Barratt impulsiveness scale 11th Version
EDE-Q 6.0: Eating disorder examination-questionnaire 6.0
BDI: Beck depression inventory
BAI: Beck anxiety inventory
DSM-5: The diagnostic and statistical manual 5th edition
BMI: Body mass index
M.I.N.I.: Mini-international neuropsychiatric interview
CI: Confidence interval
p: P-value
F: F-value
β: Beta-value
SE: Standard error
Partial η²: Partial eta-squared
