# Supplementary material for: The correlation between anorexia nervosa and childhood traumatic experience: the mediating role of impulsivity
Source: J Eat Disord. 2026 Feb 26;14:74. doi: 10.1186/s40337-026-01557-2 (PMC13041135; doi:10.1186/s40337-026-01557-2)
Supplement: Supplementary file 1 — Supplementary Material 1. [file 40337_2026_1557_MOESM1_ESM.docx]

Supplementary Table 1. Comparison of childhood traumatic experience between AN-R group, AN-BP group and HC groups with age and BMI controlled (ANCOVA)

|  | AN-R  (n=76) | AN-BP  (n=81) | HC  (n=124) | *F* | *p* | partial η² | Pairwise comparison *p*-values | | |
| --- | --- | --- | --- | --- | --- | --- | --- | --- | --- |
|  |  |  |  |  |  |  | AN-R Vs AN-BP | AN-R Vs HC | AN-BP Vs HC |
| ETI-SF  Total score | 3.14±  3.01 | 5.56±  4.85 | 4.22±  3.53 | 6.711 | 0.001 | 0.056 | 0.018 | 1.000 | 0.004 |
| General trauma | 0.56±  1.17 | 1.02±  1.73 | 1.21±  1.57 | 0.993 | 0.372 | 0.009 | 0.673 | 0.605 | 1.000 |
| Physical trauma | 1.28±  1.45 | 1.84±  1.79 | 1.57±  1.42 | 2.837 | 0.061 | 0.024 | 0.258 | 1.000 | 0.096 |
| Emotional abuse | 1.11±  1.47 | 2.32±  1.94 | 1.25±  1.45 | 14.665 | 0.000 | 0.114 | 0.005 | 0.235 | 0.000 |
| Sexual trauma | 0.18±  0.11 | 0.39±  0.10 | 0.18±  0.77 | 1.704 | 0.184 | 0.015 | 0.387 | 1.000 | 0.386 |

**Note**. All means presented are adjusted marginal means (± SEM) derived from analysis of covariance (ANCOVA) controlling for age and BMI. Raw descriptive statistics are provided in Table 1 of the main text.
